# Enhanced insulin sensitivity variability in the first 3 days of ICU stay: implications for tight glycemic control

**DOI:** 10.1186/cc9808

**Published:** 2011-03-11

**Authors:** JG Chase, AJ Le Compte, S Penning, KT Moorhead, P Massion, JC Preiser, CG Pretty, GM Shaw, T Desaive

**Affiliations:** 1 University of Canterbury, Christchurch, New Zealand; 2 University of Liege, Belgium; 3 CHU de Liege, Belgium; 4 Erasme University Hospital, Brussels, Belgium; 5 Christchurch Hospital, Christchurch, New Zealand

## Introduction

Effective tight glycemic control (TGC) can improve outcomes, particularly in cardiovascular surgery, but is difficult to achieve. Variability in insulin sensitivity/resistance resulting from the level and evolution of stress response, particularly early in a patient's stay, can lead to hyperglycemia and variability, which are associated with mortality. This study quantifies the daily evolution of the variability of insulin sensitivity for cardiovascular surgical and all other ICU patients.

## Methods

Retrospective analysis of SPRINT TGC study data. Model-based insulin sensitivity (SI) was identified hourly from data. Hour-to-hour percentage changes in SI were assessed for cardiovascular surgical (CVS) patients (*n *= 76) and all other, noncardiovascular surgery (Non-CVS) patients (*n *= 317). Results are compared for days 1, 2, 3 and days 4 onward. Cumulative distribution functions (CDFs), median values, and interquartile points (25th and 75th percentiles) are used to assess differences between groups and their evolution over time.

## Results

CVS patients are more variable than Non-CVS patients on days 1 to 2 (*P *< 0.005) and similar on days 3 and 4 onward (*P *≥ 0.13). Variability declines by day. CVS and Non-CVS patients are both more variable on each of days 1 to 3 than the overall day 4 onward values (*P *< 0.005). At the interquartile percentiles, CVS patients are 1.4 to 2.0 times more variable than Non-CVS patients on day 1, 1.40 to 1.44 times on day 2, and 1.1 to 1.2 times on day 3, but identical (< 1.1x difference) for day 4 onward. Absolute SI increases daily for both groups, and the difference between groups shrinks from 33% to 12% over days 1 to 3 and is 4% on day 4 onward (*P *< 0.005 for all). Glycemic control was equivalent for both groups (*P *> 0.05) and thus these results were not due to differences in TGC achieved, but patient-specific factors instead.

## Conclusions

All ICU patients exhibit greater insulin sensitivity variability over days 1 to 3, and cardiovascular surgery patients are more variable than others. Clinically, the results imply that TGC patients, especially cardiovascular surgery patients, will require greater measurement frequency, reduced reliance on insulin, and more explicit specification of carbohydrate nutrition in days 1 to 3 to safely minimise glycemic variability and maximise control for best outcome.

